# Systemic Capillary Leak Syndrome as a Rare, Potentially Fatal Complication of COVID-19: A Case Report and Literature Review

**DOI:** 10.7759/cureus.42837

**Published:** 2023-08-02

**Authors:** Shotaro Naito, Hiroyuki Yamaguchi, Noboru Hagino

**Affiliations:** 1 Department of General Internal Medicine, Fukushima Medical University, Fukushima, JPN; 2 Department of Rheumatology, Teikyo University Chiba Medical Center, Chiba, JPN

**Keywords:** compartment syndrome, hypoalbuminemia, hemoconcentration, hypotension, covid-19 complication, clarkson’s disease, systemic capillary leak syndrome

## Abstract

Systemic capillary leak syndrome (SCLS), also known as Clarkson’s disease, is a rare and potentially lethal condition characterized by hypotension, hemoconcentration, and hypoalbuminemia; however, the cause of SCLS is still uncertain. We present the case of a 62-year-old male with flu-like symptoms who presented to the emergency department with shock. Initial evaluation revealed hemoconcentration, hypoalbuminemia, acute kidney failure, and positive polymerase chain reaction (PCR) for severe acute respiratory syndrome coronavirus 2 (SARS-CoV-2). Despite aggressive fluid resuscitation, the shock persisted, and the patient’s condition deteriorated. After ruling out ischemia and septic shock, the patient was diagnosed with coronavirus disease 2019 (COVID-19)-associated SCLS. Treatment with remdesivir and intravenous immunoglobulin (IVIG), along with the restoration of intravascular volume, led to the gradual improvement of the patient’s condition. The patient experienced pulmonary edema, which was managed by correcting the fluid balance through continuous hemodiafiltration. Eventually, the patient recovered without any residual organ complications. SCLS is often misdiagnosed because of its rarity and non-specific symptoms. Accurate diagnosis and understanding of the disease’s pathophysiology are crucial for effective management. This report contributes to the existing literature by presenting a case of COVID-19-associated SCLS and emphasizes the need for further research on its occurrence and outcomes.

## Introduction

Idiopathic systemic capillary leak syndrome (iSCLS), also known as Clarkson’s disease, is a rare, potentially fatal disease. Its diagnosis is based on the presence of the clinical triad of hypotension, hemoconcentration, and hypoalbuminemia, termed the three Hs, with the exclusion of alternative etiologies for these symptoms. iSCLS exhibits a three-phase progression, commencing with the prodromal phase, followed by the leakage phase, and culminating in the recovery phase. During the prodromal phase, patients may present with flu-like symptoms, which are followed by refractory hypovolemic shock and anasarca owing to fluid extravasation, which may lead to compartment syndrome and multi-organ failure in severe cases. After several days of the leakage phase, extravasated fluids are reabsorbed into the vessels, which can often result in pulmonary edema during the recovery phase [1]. The management of systemic capillary leak syndrome (SCLS) primarily involves an appropriate systemic approach, although some reports have demonstrated the efficacy of intravenous immunoglobulin (IVIG) [2]. Despite ongoing investigations, the underlying etiology of SCLS remains unclear, although viral infections, including coronavirus disease 2019 (COVID-19), are considered a significant secondary trigger [3]. Severe acute respiratory syndrome coronavirus 2 (SARS-CoV-2) has emerged as a ubiquitous ailment with a global footprint. Infection- and vaccine-triggered SCLS have been documented in a limited cohort and warrant acknowledgment as an infrequent yet possibly lethal manifestation of a common affliction [4-17]. In this regard, we present a case of SCLS that may be associated with COVID-19 and provide a comprehensive examination of this case through a critical analysis of previously documented cases.

## Case presentation

A 62-year-old male with a history of systemic hypertension and vasospastic angina pectoris presented at our emergency department with dizziness accompanied by malaise and anorexia. He had previously experienced fever, chills, and urinary difficulty three days before arriving. The patient had no documented hypersensitivity and had not been recently initiated on new medications. The patient had received two doses of the SARS-CoV-2 vaccine (mRNA-1273 vaccine, Moderna) two years preceding the current evaluation. Physical examination revealed a body temperature of 36.1°C, blood pressure of 121/81 mmHg, pulse rate of 98 beats per minute, respiratory rate of 24 breaths per minute, and oxygen saturation of 96% on ambient air. Although the abdomen appeared soft and flat, diffuse tenderness was primarily observed in the lower quadrant, with coldness of the lower limbs also noted. The remaining physical examination results were unremarkable. Laboratory findings revealed hemoconcentration (hemoglobin: 17.3 g/dL), hypoalbuminemia (serum albumin: 1.9 g/dL), acute kidney failure (4.1-fold increase in serum creatinine over baseline and anuria exceeding six hours), and a positive polymerase chain reaction (PCR) for SARS-CoV-2 (cycle threshold {Ct} value: 45). Additional laboratory data are presented in Table 1. Electrocardiography revealed sinus tachycardia, and chest radiographs and CT scans were unremarkable.

**Table 1:** Pre-admission and in-patient laboratory findings in our case. ND: no data

	Two weeks earlier	Hospital day 1	Hospital day 2	Hospital day 5	Hospital day 21	Reference range
Leukocytes (/μL)	6700	7200	12000	8700	5300	3900-9800
Hemoglobin (g/dL)	14.3	17.3	21.1	10.3	10.1	13.5-17.6
Platelets (×10000/μL)	26.4	16.9	20.9	6.6	30.4	13.1-36.2
Albumin (g/dL)	4.0	1.9	2.1	1.4	3.2	3.8-5.2
Creatinine (mg/dL)	0.63	1.50	2.76	1.92	1.19	0.61-1.04
Creatinine kinase (U/L)	220	119	314	27020	249	62-287
Lactate (mmol/L)	ND	ND	7.47	1.29	ND	0.36-1.40
C-reactive protein (mg/dL)	<0.3	0.6	1.1	10.6	0.6	0.0-0.3

Given the presence of fever with shivering and chills, in conjunction with the urinary symptoms, we suspected urinary tract infection and initiated ceftriaxone antimicrobial therapy. The patient became severely hypotensive on the day of hospital admission. Subsequent echocardiography revealed no signs of cardiogenic shock. The patient received supportive management with fluids and catecholamines, with suspected septic shock as the underlying etiology. Despite receiving more than 10 L of Ringer’s solution, the shock persisted, and hemoconcentration (hemoglobin: 21.1 g/dL) developed. The patient’s general condition continued to deteriorate, and he experienced diffuse abdominal pain, generalized edema, and myalgia. A contrast-enhanced examination of the abdominal vessels was performed, owing to suspected intestinal ischemia; however, no findings suggestive of ischemia were observed. Subsequently, the patient was transferred to the intensive care unit and received systemic management. However, the circulatory dynamics continued to deteriorate. On the third day of admission, he developed pulseless electrical activity, which led to cardiopulmonary arrest. Three cycles of cardiopulmonary resuscitation were administered, and the patient’s heartbeat was restored. The patient was intubated and ventilated, and continuous hemodiafiltration was initiated because of persistent anuria. The patient underwent targeted temperature management for 48 hours, during which no unfavorable neurological prognostic indicators were observed. Blood and urine cultures obtained upon admission yielded negative results. Therefore, septic shock was excluded. Repeated SARS-CoV-2 PCR revealed a decreased Ct value of 18, indicating an increased viral burden. The patient was diagnosed with COVID-19-associated SCLS based on the progression of hypotension, hemoconcentration, and hypoproteinemia despite receiving extensive fluid therapy, with the exclusion of other differential diagnoses.

**Figure 1:**
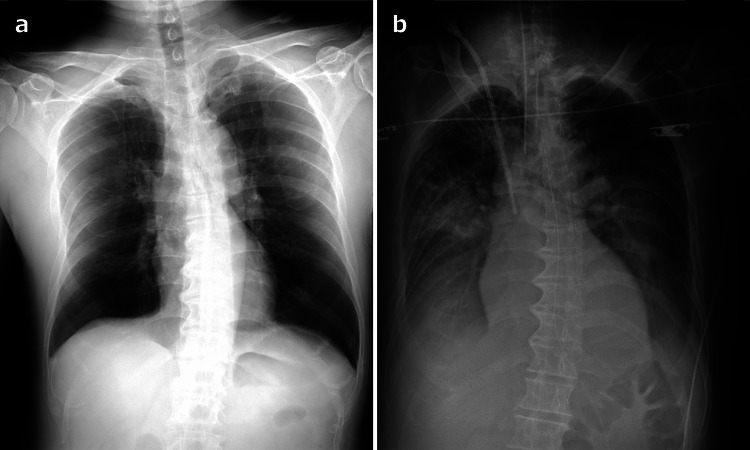
Chest radiographs obtained during hospitalization. (a) Initial chest radiograph obtained on admission was unremarkable. (b) Chest radiograph obtained on the sixth day of hospitalization showed pulmonary edema and pleural effusion.

Therapeutic interventions for the primary disease and SCLS included the administration of remdesivir and intravenous immunoglobulin. On the sixth day of hospitalization, the patient’s intravascular volume was restored, leading to a considerable reduction in intravenous infusion requirements and the gradual tapering of catecholamines. The patient also experienced an upsurge in oxygen demand due to pulmonary edema associated with refilling (Figure 1 shows chest radiographs). Following the correction of the fluid balance through continuous hemodiafiltration, the patient was extubated and gradually weaned off the ventilator on the eighth day of hospitalization, because pulmonary edema was resolved by oxygenation. Significant urination occurred spontaneously, facilitating the cessation of continuous hemodiafiltration therapy. Renal function improved to baseline with no residual organ complications. Paraproteins have been identified in approximately 70% of patients diagnosed with iSCLS and are recognized as underlying pathogenic factors [1]. In our patient, the immunofixation of the recovered plasma revealed the presence of immunoglobulin G (IgG)-λ paraproteins. The patient recovered without neurological sequelae and was discharged after rehabilitation.

## Discussion

SCLS is an extremely rare and potentially life-threatening condition. The underlying cause remains unknown, but it is believed to involve abnormal vascular permeability leading to hypotension, hemoconcentration, and hypoproteinemia. Paraproteins are detected in around 70% of patients, suggesting a potential association with hematologic conditions such as monoclonal gammopathy of undetermined significance [2]. SCLS can occur as a secondary response to various triggers, including viral infections. COVID-19-associated SCLS is of particular interest due to its rarity and potential lethality. Depending on the trigger, secondary SCLS may follow a course that is distinct from that of iSCLS [3]. Therefore, it is essential to identify the similarities and differences between COVID-19-associated SCLS and iSCLS. In our assessment, we focused on identifying the similarities between COVID-19-associated SCLS and iSCLS.

To achieve this objective, we conducted a comprehensive review of the literature published from 2020 to 2022, focusing on case reports of COVID-19-associated SCLS in adults (≥18 years). Our analysis included patient characteristics, symptoms, laboratory data (hemoglobin, serum albumin, serum creatinine, lactate, and paraprotein levels), acute treatment, and prognosis. We screened 23 reports from PubMed using the Medical Subject Heading (MeSH) terms “COVID-19” and “systemic capillary leak syndrome” and finally selected 14 reports that specifically described COVID-19-associated SCLS [4-17].

Epidemiology

To investigate the epidemiology of COVID-19-associated SCLS, a total of 18 cases were included in our review, with 10 cases associated with COVID-19 and eight cases following vaccination (Table 2). The average age of the patients was 49.5 years, and there was no notable difference in clinical characteristics between both sexes. The epidemiological distribution in our study was similar to those of iSCLS [1].

**Table 2:** Patient demographics and clinical features of SCLS associated with COVID-19, as outlined in the literature. SCLS, systemic capillary leak syndrome; COVID-19, coronavirus disease 2019; IVIG, intravenous immunoglobulin; mPSL, methylprednisolone; ND, no data

Article	Age (years)	Sex	Trigger	Prior SCLS	IVIG	Steroid	Outcome	Hemoglobin (g/dL)	Albumin (g/dL)	Creatinine (mg/dL)	Lactate (mmol/L)
Araki et al. [4]	53	Female	Vaccine	No	No	mPSL	Survived	20.8	0.8	ND	ND
Pineton de Chambrun et al. [5]	45	Female	Infection	Yes	No	No	Died	19.1	ND	ND	ND
Case et al. [6]	63	Male	Infection	No	No	No	Died	21.6	3.5	2.34	4.9
Cheung et al. [7]	59	Female	Infection	No	Yes	No	Died	17.1	3.8	0.95	3.7
Cheung et al. [8]	36	Male	Infection	Yes	No	No	Died	25	ND	ND	9.2
Choi et al. [8]	38	Male	Vaccine	No	No	No	Died	22.7	3.3	2	5.4
Knox et al. [9]	48	Female	Infection	No	Yes	No	Survived	16.4	3	ND	ND
Robichaud et al. [10]	66	Male	Vaccine	Yes	No	No	Survived	22.4	2.8	1.33	3.8
Inoue et al. [11]	40	Female	Vaccine	No	Yes	mPSL	Survived	24.1	1.8	1.92	3.91
Concistrè et al. [12]	58	Male	Infection	Yes	Yes	No	Survived	17.3	3	1.3	2.2
Novotná et al. [13]	42	Male	Infection	No	No	No	Died	19.8	4	1.08	4.4
Lacout et al. [14]	38	Male	Infection	No	No	No	Survived	24.9	1.6	1.47	5.7
Beber et al. [15]	55	Female	Infection	No	Yes	No	Survived	16.8	3.5	0.8	ND
Buj et al. [16]	38	Male	Vaccine	No	No	No	Survived	23.6	1.7	3.8	6.88
Matheny et al. [17]	68	Female	Vaccine	No	Yes	No	Died	20.1	1.1	2.59	10.9
Matheny et al. [17]	46	Female	Vaccine	Yes	No	Stress dose	Survived	23.3	2	1.5	7.7
Matheny et al. [17]	36	Male	Vaccine	No	No	Stress dose	Survived	19.9	2.3	2.4	10.9
Our case	62	Male	Infection	No	Yes	No	Survived	17.3	2.4	1.5	7.47
Mean	49.50							20.68	2.54	1.78	6.22
Standard deviation	10.80							2.84	0.94	0.77	2.63

Clinical manifestations

The clinical manifestations of COVID-19-associated SCLS included flu-like symptoms in 78% of all cases and gastrointestinal symptoms in 50% of all cases (Table 3). Dizziness or syncope was reported in 27.8% of all cases. Druey and Parikh reported flu-like symptoms in up to three-quarters of patients with iSCLS, and 64.4% of patients experienced dizziness, 52.5% experienced nausea and vomiting, and 35.6% had abdominal pain [1], consistent with the findings of the present study.

**Table 3:** Primary clinical manifestations of patients with SCLS associated with COVID-19, as reported in the literature. Flu-like symptoms: fever, general malaise, and upper respiratory tract symptoms. Gastrointestinal symptoms: abdominal pain, nausea, vomiting, and diarrhea SCLS, systemic capillary leak syndrome; COVID-19, coronavirus disease 2019

Primary clinical manifestations	Number of patients	Percentage (%)
Flu-like symptoms	14	77.8
Gastrointestinal symptoms	9	50.0
Dizziness or syncope	5	27.8
Myalgia	4	22.2
Arthralgia	2	11.1
Edema	2	11.1
Seizure	1	5.6
Chest pain	1	5.6
Back pain	1	5.6
Difficulty in urination	1	5.6

Laboratory data

Laboratory data revealed hemoconcentration, hypoalbuminemia, elevated creatinine, and increased lactate levels (Table 2). Paraproteins were detected in 50% of all patients; IgG-kappa was the predominant paraprotein detected. These data were comparable to those reported in previous literature reviews of iSCLS [1,18,19].

Treatment

Regarding treatment, supportive measures such as circulation management formed the basis of acute-phase management, with limited evidence supporting other therapeutic options (Table 2). IVIG is occasionally used during the acute phase since it can neutralize autoantibodies and inflammatory cytokines [2]. It was also the most frequently used medication according to our review.

Prognosis

The prognosis of COVID-19-associated SCLS was investigated by examining patient outcomes in all 18 cases, revealing an overall mortality rate of 39% (Table 4). Additionally, the mortality rate was slightly higher in the infected group than in the vaccinated group. These results may reflect differences in the amount of antigen exposure.

**Table 4:** Outcomes of patients with SCLS associated with COVID-19, as reported in the literature. SCLS, systemic capillary leak syndrome; COVID-19, coronavirus disease 2019; n, number of patients

SCLS trigger	Survival, n (%)	Death, n (%)	Total, n
Infection	5 (50%)	5 (50%)	10
Vaccine	6 (75%)	2 (25%)	8
Overall	11 (61%)	7 (39%)	18

The number of COVID-19-associated SCLS cases reported within the two-year period of 2020-2022 was higher than that of other secondary SCLS cases [3]. Inflammatory cytokine flares observed in severe COVID-19 and SCLS suggest a potential predisposition to SCLS complications in COVID-19 patients. Given the expected global impact of COVID-19, with recurrent outbreaks and the emergence of mutant strains, it is crucial for clinicians to be aware of SCLS as a rare yet serious early complication. Our literature review reveals that COVID-19-associated SCLS has a clinical course similar to that of iSCLS. We believe that this distinct clinical presentation could aid in the early detection of SCLS.

## Conclusions

We present a case of SCLS associated with COVID-19. SCLS is a potentially fatal condition characterized by hypotension, hemoconcentration, and hypoalbuminemia. The complexities involved in diagnosing SCLS, owing to its non-specific symptomatology, often result in underdiagnosis or misdiagnosis. COVID-19 represents one of the most prevalent infectious diseases, and as sporadic outbreaks will likely continue in the future, it is crucial for clinicians to recognize SCLS as a rare yet potentially life-threatening complication of COVID-19.
